# Deep Metric Learning-Based Strawberry Disease Detection With Unknowns

**DOI:** 10.3389/fpls.2022.891785

**Published:** 2022-07-04

**Authors:** Jie You, Kan Jiang, Joonwhoan Lee

**Affiliations:** Artificial Intelligence Lab, Department of Computer Science and Engineering, Jeonbuk National University, Jeonju, South Korea

**Keywords:** deep metric learning, unknown disease detection, strawberry disease detection, *K*-nearest neighbor, open set recognition

## Abstract

There has been substantial research that has achieved significant advancements in plant disease detection based on deep object detection models. However, with unknown diseases, it is difficult to find a practical solution for plant disease detection. This study proposes a simple but effective strawberry disease detection scheme with unknown diseases that can provide applicable performance in the real field. In the proposed scheme, the known strawberry diseases are detected with deep metric learning (DML)-based classifiers along with the unknown diseases that have certain symptoms. The pipeline of our proposed scheme consists of two stages: the first is object detection with known disease classes, while the second is a DML-based post-filtering stage. The second stage has two different types of classifiers: one is softmax classifiers that are only for known diseases and the *K*-nearest neighbor (*K*-NN) classifier for both known and unknown diseases. In the training of the first stage and the DML-based softmax classifier, we only use the known samples of the strawberry disease. Then, we include the known (*a priori*) and the known unknown training samples to construct the *K*-NN classifier. The final decisions regarding known diseases are made from the combined results of the two classifiers, while unknowns are detected from the *K*-NN classifier. The experimental results show that the DML-based post-filter is effective at improving the performance of known disease detection in terms of mAP. Furthermore, the separate DML-based *K*-NN classifier provides high recall and precision for known and unknown diseases and achieve 97.8% accuracy, meaning it could be exploited as a Region of Interest (ROI) classifier. For the real field data, the proposed scheme achieves a high mAP of 93.7% to detect known classes of strawberry disease, and it also achieves reasonable results for unknowns. This implies that the proposed scheme can be applied to identify disease-like symptoms caused by real known and unknown diseases or disorders for any kind of plant.

## Introduction

There has been much research into plant disease detection based on the deep object detection technique, and substantial advancements have been achieved in this field (Zhao et al., 2019). The object detection models for plant diseases have been developed in two directions: One is for better precision (Ren et al., 2015; Lin et al., 2017b; Tan et al., 2020) while the other is for faster response (Redmon and Farhadi, 2018; Zhang et al., 2018; Bochkovskiy et al., 2020). There are now many off-the-shelf object detection models that can be chosen for plant disease detection for a specific purpose (Xiao et al., 2021; Dananjayan et al., 2022).

In constructing a plant disease detector, researchers collect samples of known diseases and then successfully train a selected object detection model using these samples. However, there may be disease-like symptoms in the inference process that are not actually from the known diseases. One of the confidence levels for the predefined disease classes might be maximum but with a low value, which means that it can produce false detection, or just miss detection according to the detection threshold. To reduce the false detection rate, the detection threshold can be increased, but the real disease with obscure symptoms might be missed. This is an undesirable situation that leads to a large number of either false or missed detections depending on the detection threshold.

Open-set detection (Bastan et al., 2019; Fehérvári and Appalaraju, 2019; Mahdavi and Carvalho, 2021) could solve this problem, as it discerns the unknown diseases as they are in the inference process, although only known diseases are taken care of in the training process. Unfortunately, the technology is not yet mature enough to be practically utilized for fine-grained plant disease detection. The state-of-the-art performance is not that good, even for coarse-grained tasks of distinct objects that look different.

Another alternative method is the post-filtering approach that effectively reduces the erroneous detections involved in the detection process. Many post-filtering schemes can be chosen, but we selected DML-based classifiers (Li and Tian, 2018; Kaya and Bilge, 2019) to be used for known and known unknown diseases. DML produces the feature space in which each cluster of the class becomes compact by reducing the intra-cluster distances and increasing the inter-cluster distances.

Our proposed scheme is similar to the object detection of plant disease followed by simple post-filtering, but the prepared unknown samples are used to classify ambiguous samples into an unknown category. The post-filtering stage has two different types of classifiers: softmax classifiers for only known diseases and the *K*-NN classifier for known and unknown diseases. In training the first stage of the object detection model and the DML-based softmax classifier, we only used known samples of the strawberry disease. Then, the known unknown training samples are included to construct the *K*-NN classifier. The final decisions for known diseases are made based on the combined results of the two classifiers, while unknowns are detected solely from the *K*-NN classifier. Table 1 summarizes the data type used to train the building blocks and their decisions in the inference process of our proposed scheme. Note that the DML-based post-filter can be used as a separate ROI classifier if the disease-like symptoms are manually annotated, as opposed to the automatic detection in the first stage. Therefore, the technology in our scheme can be exploited for both the detection and classification of plant diseases.

**TABLE 1:** Proposed data type scheme for known and unknown disease detection.

	Type of disease data	First object detection stage	Second stage DML-based post-filter		
			Softmax	*K*-NN	Combined**
Training	Known	Used	Used	Used	Not used
	Unknown	Not used	Not used	Used	Not used
Inference	Known	Detected	Classified	Classified	Classified
	Unknown	Possibly detected*	Not classified	Classified	Not classified

*Disease-like symptom can be detected in the first object detection model, but it is determined by the K-NN classifier.**This stands for the final decision of the combined softmax and K-NN classifiers for known disease.

In the experiment, we adapt Faster R-CNN with Feature Pyramidal Network (FPN) for the object detection model and margin triplet loss for DML. To verify our scheme, we constructed a strawberry disease dataset and used it for the experiment. The contributions of this study can be summarized as follows:

(1) This study proposes a practical solution for detecting known and partly known unknown plant diseases that provide good detection performance. It achieves approximately 93.7% of mAP to known classes of strawberry disease, and it also achieves reasonable results for unknowns of real field data.
(2) The proposed scheme consists of two stages: the object detection stage and the DML-based post-filter stage. The object detection model can be freely chosen according to the design requirement because it can be separated from the following DML-based post-filter. In addition, the DML-based post-filter can be separated from the first stage, and it can also be exploited for the ROI-based classifier of known and unknown diseases. The separate DML-based *K*-NN classifier provides high recall and precision for both known and known unknown diseases.

## Related Works

The proposed scheme consists of two consecutive stages of an object detection model, followed by add-on post-filtering. This section reviews the related works to our scheme, which include object detection for monitoring plant disease, DML to separate clusters of classes, and *K*-NN classifier for known unknown detection.

### Object Detection Models for Plant Disease Monitoring

As mentioned previously, various object detection models are available for plant disease monitoring. They have been developed to achieve two objectives: better accuracy and higher speed. Faster R-CNN (Ren et al., 2015; Lin et al., 2017b) is a 2-stage model that is relatively slow but accurate. On the other hand, the YOLO family and SSD (Zhang et al., 2018) start from a single stage with detection performance that is fast but less accurate. However, there have been continuous developments aiming for better accuracy while sacrificing speed. For example, the recent version of the YOLO family (Redmon et al., 2016; Redmon and Farhadi, 2017, 2018; Bochkovskiy et al., 2020) provides many design options according to different requirements. Moreover, a recent transformer model (Carion et al., 2020) for object detection has been announced, and it is ready to be further developed to compete with Convolutional Neural Network (CNN)-based models. In addition, diverse models have been developed to meet the needs of various applications, even if there are few application examples for plant disease detection (Lin et al., 2017a; Tan et al., 2020).

For plant disease detection, a model with better speed could be required, such as light YOLO v.5. A mobile robot can capture plant images in a greenhouse, and the board embedded in the robot can help automatically identify disease symptoms in the field. On the other hand, the captured images can be transmitted to a remote cloud site of a high-performance computing facility to be precisely scrutinized using an accurate but slow model. In this situation, Faster R-CNN or its variants, such as cascaded Faster R-CNN, would be a better choice. Note that the classification approach for monitoring diseases is hard to automatize (Kim et al., 2021); this is because the image-containing symptoms of the disease should be manually located to take pictures and then fed into the classification-based monitoring system. However, it is still an important way to identify known and unknown diseases or disorders. Kim et al. (2021) and Liu and Wang (2021) provide excellent reviews of deep learning-based disease detection and classification models.

### Post-filtering and Deep Metric Learning

The post-filtering approach is a practical way to improve detection accuracy, and it can be added to plant disease detection. Because the additional post-filter can reduce false detections, the confidence threshold of the detection stage can typically be lowered to increase the recall, even if that increases the number of false detections. Fuentes et al. (2020) adapted the idea to their one-*versus*-all post-filtering approach in tomato disease detection, while Kim et al. (2021) shared a similar idea in their cascaded Faster R-CNN for strawberry disease detection.

In this study, we propose the use of DML to build a low-dimensional feature space of known disease classes, where the clusters are well separated, by increasing the inter-cluster distances while reducing the intra-cluster distance (Kaya and Bilge, 2019). Furthermore, Ji et al. (2021) proposed a framework in which the features are learned by a deep learning feature extractor and WDM-tSNE is applied to accurately cluster the feature space of plant disease. In general, metric learning is done to obtain a proper metric for classifying objects, which captures a mapping function from visual objects to a low-dimensional embedded feature space with respect to a predefined distance metric, such as Euclidian or L1 distance. There are two different metric learning structures with different losses: one is the Siamese structure that uses contrastive loss (Chopra et al., 2005) and the other is the triplet structure with triplet loss (Schroff et al., 2015). Janarthan et al. (2020) have adapted the former structure to citrus disease classification. In our scheme, we choose the latter triplet structure. The essence of the DML in our scheme is to obtain a mapping that will separate clusters of known classes well in the feature space to make sufficient room for the known unknown diseases. Better classification performance for known diseases can be obtained by applying the softmax classifier to the embedded features from the metric learning. However, for the unknowns, we used the *K*-NN classifier based on the DML-embedded features that could be lost or falsely detected when only the object detection is applied. Although the object classifier after the object detection produces better performance, it is difficult to include the known unknowns, because there could be a huge set of unknown unknowns that are only experienced in the inference process. In other words, previous methods could not well expect the unknown unknowns in the training process.

### Open World Setting for Unknown Disease Recognition

Significant progress has been made with machine intelligence, which is another technique for continual and life-long learning for open-world recognition, even if it is premature for practical applications, especially fine-grained tasks (Schlachter et al., 2019a,b, 2020; Geng et al., 2020). In the most general problem settings of the open world, no type of unknown can be contained in the training dataset, that is, it only appears in the test environment. Joseph et al. (2021) identify the open-world detection problem in 3-dimensional space, where one axis is the direction of increasing problem difficulty, one axis is the direction of open-set learning, and the last axis is incremental learning. In terms of the first axis of problem difficulty, open-set identification is more difficult than classification alone. However, if there is no prior assumption of unknowns, as is the case in the traditional open-set recognition problem setting, then the resulting state-of-the-art classification performance is not that good. For example, the state-of-the-art performance for easy MNIST, SVHN, and CIFAR-10 dataset exceeds 90%, but for difficult CUB and ImageNet dataset does not reach 90% in terms of AUROC (Vaze et al., 2021). In open object detection, which is a much harder problem than classification, the technology is far from being practically applicable for difficult plant disease detection. Because incremental learning (Parisi et al., 2019) for continual and life-long learning (Parisi et al., 2019) is beyond the scope of our work, it is not reviewed in this article, although it is related to open-set recognition.

In this article, we release the constraints on the rigorous open-set problem setting. For example, we do not know the name of the disease for samples, but they certainly exhibit similar disease-like symptoms that may have originated from diseases or disorders. Compared to the samples of major diseases, such samples look diverse and the frequency of similar objects is rare. One point that we want to emphasize is that the classifier performance of the closed set data is positively correlated with that of the open-set data (Parisi et al., 2019). In our scheme, DML tries to make a better classifier for the closed disease dataset while simultaneously leaving large empty room to locate unknowns.

## Methods

**FIGURE 1:**
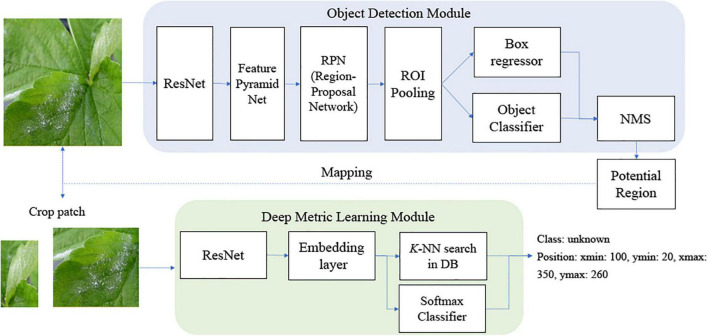
Structure of overall scheme for inference.

Figure 1 shows a schema of the proposed scheme. Our scheme is divided into two stages: the object detection module and the deep metric learning module. In the training, the object detection module can be trained with known disease samples to find as many potential known disease positions with the object classifier as possible. Then, the feature embedding of the post-filter is trained by DML to separate the clusters of known classes well. In the deep metric learning module, we cannot consider the unknown disease-like samples, so the training of the post-filter is identical to that of the conventional method of object detection and its refinement. Note that we enlarged the bounding boxes of the object detection results and sent for post-filter training; this is done to allow for dislocation of the object detection results and to include more context information around disease. Then, the embedded features of bounding boxes of known diseases are extracted from the DML-learned network to build the softmax classifier. Once the DML-learned network and softmax classifier training is finished, the weight is frozen and DML-embedded features from known and known unknown samples are used to build the *K*-NN classifier.

In the inference process, known and unknown disease samples are fed into the trained object detector. Then, the extended bounding box around the symptom is given to extract DML-trained features to be categorized by the softmax and *K*-NN classifiers. In this study, the softmax classifier is only concerned with known diseases, while the *K*-NN classifier deals with both known diseases and unknowns. The overall classification category of known diseases can be made by the combined decision of softmax and *K*-NN classifiers.

### Object Detection Model

**FIGURE 2:**
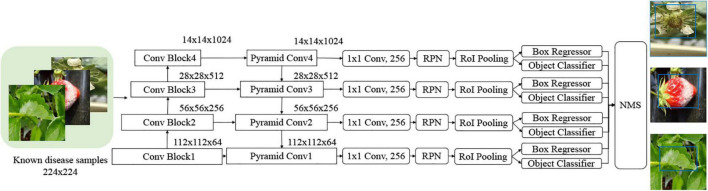
Feature pyramidal network (FPN)-based Faster R-Convolutional Neural Network (CNN) for potential disease detection.

As described in the previous section, there have been diverse object detection technologies for plant disease monitoring. In our scheme, we choose FPN-Based Faster R-CNN for accurate detection. According to the open-set object detection, it provides the best accuracy based on standard protocol (Dhamija et al., 2020). Note that our scheme cannot detect unknown unknowns, because these are inevitably ignored in the training of the building blocks of our scheme. The object classifier in the object detection module distinguishes the known diseases from the background and produces the classification probability for knowns. Figure 2 shows the conventional FPN-Based Faster R-CNN, which can detect various sizes of objects due to the exploitation of the pyramidal feature structure (Lin et al., 2017b). In this study, we want to emphasize that a low detection threshold would be better so as not to ignore the disease-like symptoms that are from unknown diseases or disorders. The size of the input image was 224 × 224 pixels to fit the CNN backbone. The number of diseases in the object detection stage was eight, including an angular leafspot, anthracnose (fruit rot, runner), blossom blight, gray mold (fruit), leaf spot, and powdery mildew (fruit, leaf). Note that some diseases show symptoms at different parts, and these are treated as different categories, because the part images are quite different.

### Deep Metric Learning for Embedded Features

Our scheme chooses the ResNet50 network with margin triplet and cross-entropy losses for DML. The embedded features are used to refine the softmax classifier. In general, there are many false detections of normal leaf, fruit, flower, and runner as one of the diseases in the first stage of object detection. In our post-filter, each one is also treated as a separate class for training DML. The false detection of normal parts as diseases can be corrected in the DML-based classifiers. Therefore, we have considered 12 known classes in the DML-learning (eight known diseases and four normal parts).

There are two losses involved in the DML of margin triplet loss for embedded features and cross-entropy loss for the softmax classifier. The margin triplet loss is defined as Schroff et al. (2015):


(1) $$L_{\mathrm{tuplet}}=\max\{d\left(f\left(x_{a}\right),f\left(x_{p}\right)\right)-d\left(f\left(x_{a}\right),f\left(x_{n}\right)\right)+\mathrm{margin}\},0)$$

where,


(2) $$d\,\left(x_{i},x_{j}\right)=\frac{x_{i}\cdot x_{j}}{\max\,\left({||x_{i}||}_{2}\cdot {||x_{j}||}_{2},\varepsilon \right)}$$

**FIGURE 3:**
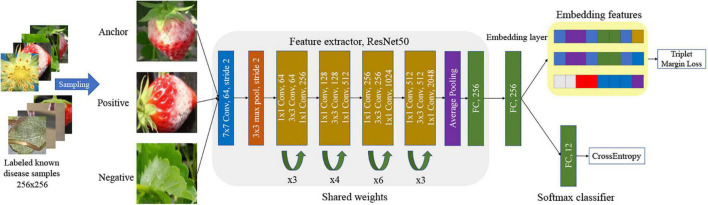
Triplet network and loss with softmax classifier.

In Eq. (1), *f*(*x*_*a*_), *f*(*x*_*p*_), and *f*(*x*_*n*_), respectively, represent the features of anchor, positive, and negative image samples after mapping *f*(), from the network in Figure 3. Here, *d*() is the Euclidian distance. The value of the margin was set to 0.01, andεwas 1e^–8^, which is a very small value to avoid dividing by zero. The cross-entropy loss is


(3) $$L_{\mathrm{ce}}=\frac{1}{N}\,\sum_{n=1}^{N}\log\,\left(\frac{e\,x\,p\,\left(f\,\left(x_{n}\right)\right)}{\sum_{c=1}^{C}e\,x\,p\,\left(f\,\left(x_{c}\right)\right)}\right)$$

where *N* spans the size of the batch and *C* is the number of classes.

Figure 3 presents the training of the DML with the softmax classifier in our scheme. The size of the input image is 256 × 256 to meet the requirements of the first CNN layer of the shared network to obtain a proper mapping in Figure 3. Note that the extended bounding boxes from the object detection step are normalized to a uniform size. During the training, the feature extractor tries to minimize the margin triplet loss, which minimizes the Euclidian distance between a pair of the anchor and positive image, and maximizes the Euclidean distance between a pair of anchor and negative image, after trainable mapping in ResNet50. In actuality, the same triplet networks sharing the weight parameters are simultaneously learned. Finally, the dimension of the embedded features that are used for the softmax classifier, and later the *K*-NN classifier is 256. We followed the method in Schroff et al. (2015) to sample semi-hard triplets to train the network. The semi-hard samples are the subset of all triplet samples, in which the distance between negative and anchor is further from the positive and anchor, ${||f\,\left(x_{i}^{a}\right)-f\,\left(x_{i}^{p}\right)||}_{2}^{2}<{||f\,\left(x_{i}^{a}\right)-f\,\left(x_{i}^{n}\right)||}_{2}^{2}$. This is a crucial step to speed up training and ensure the network convergence.

### *K*-Nearest Neighbor (*K*-NN) Classifier for Categorizing the Diseases With Known Unknown Samples

**FIGURE 4:**
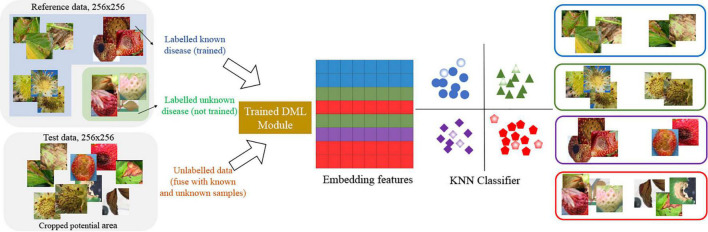
*K*-nearest neighbor (*K*-NN) classifier to categorize the disease classes with unknowns.

In the second stage of our scheme, the *K*-NN classifier (Schroff et al., 2015) is built as a lazy learner. Here, the reference data includes known and known unknown samples with normal parts for the *K*-NN classifier. As a result, the number of classes in the *K*-NN classifier is 13, consisting of eight known diseases, four normal parts, and the class for known unknowns. In the experiment, we set *K* = 13 and chose a class randomly when the tie happens on multiple majority classes. Figure 4 shows how the images are mapped into 256-dimensional embedded features and how to decide one of the class labels including unknowns in the *K*-NN classifier.

Note that there are duplicate classifiers in our scheme; one is from the softmax classifier and the other is the *K*-NN classifier. They both exploit DML-embedded 256-dimensional feature, but the softmax classifier does not take care of unknowns. As a result, there are 12 categories for the softmax classifier and one more unknown category for the *K*-NN classifier. There is no specific reason to make a different number of categories except for the fact that the softmax classifier is solely focused on known diseases to measure its performance in terms of average precision (AP) and mean AP (mAP), while the *K*-NN classifier considers both the known and unknown diseases.

The final classification of the known diseases and normal parts can be obtained by combining the two different decisions: one from the softmax classifier and the other from the *K*-NN classifier. There are typically no probabilities from the *K*-NN classifier, but we define the probability of the *j*-th class as:


(4) $$p_{j}^{K-\mathrm{NN}}=\frac{\mathrm{the}\,\mathrm{number}\,\mathrm{of}\,\mathrm{nearest}\,\mathrm{neighbors}\,\mathrm{in}\,\mathrm{class}\,j}{K}$$

(5) for⁢j∈{1,2,…,C}

In the experiment, *C=12* without the unknown class. The probability can be combined with that from the softmax output to make the final decision. We simply multiply the two probabilities and take the class that has the maximum value, as in Eq. (5):


(6) $$\mathrm{class}\,\mathrm{label}=\arg\max\left\{p_{j}^{K-\mathrm{NN}}\times p_{j}^{\mathrm{softmax}}\right\}$$

where $p_{j}^{s\,o\,f\,t\,m\,a\,x}$ denotes the output probability of the softmax classifier. Therefore, the final decision rules for known diseases and unknowns can be summarized as follows:

**Table:** 

*Rules*
1) If the *K*-NN classifier decides the image sample is unknown, it is an unknown disease.
2) Otherwise, refer to Eq. (5) to decide the proper class and probability among known. classes.

## Experimental Results

### Dataset for Experiment

For the experiments, an image dataset of strawberry diseases is constructed from the images taken by cellular phones in many greenhouses. The total number of images in the dataset is 7,230, and angular leafspot, anthracnose (fruit rot, runner), blossom blight, gray mold (fruit), leaf spot, and powdery mildew (fruit, leaf) disease images are included with normal images of flower, fruit, leaf, and runner. The disease images were taken by a cellular phone without any additional treatment to provide a more realistic appearance.

### Training Feature Pyramidal Network (FPN)-Based Faster R-Convolutional Neural Network (CNN) Object Detector for Disease Monitoring

**FIGURE 5:**
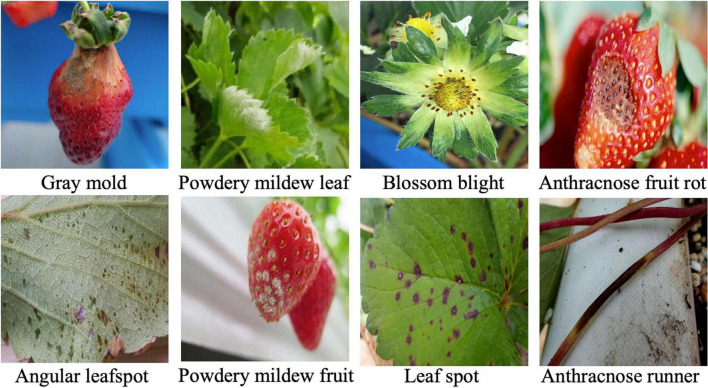
Sample images for training disease detection.

For the training, the diseases and their bounding boxes enclosing the symptoms were annotated. The number of bounding boxes for each disease used for training and testing are, respectively, listed in columns 1 and 2 of Table 2. Note that we strictly split the set of images into training and testing sets with a ratio of 4:1 (5423:1807). Table 2 only counts the number of bounding boxes. There may be more than one bounding box in an image. During the training, the online augmentation technique is applied to avoid overfitting by taking geometric transforms of horizontal/vertical flips and resizing, color jittering, blurring, and mosaicking. The total number of disease categories in this disease detection step was eight, and the results of classification were given one of the disease classes with proper bounding boxes. The training started from the weight parameters pretrained on the PlantNet in LifeCLEF 2017 dataset (Heredia, 2017), with the learning rate set to 0.002 and training for 180,000 iterations. To avoid local optimization, the learning rate was reduced by 10% at 30,000/50,000/130,000 iterations. The momentum was set to 0.9, and the stochastic gradient descent optimizer was used to minimize the difference from the ground truth. For better understanding, Figure 5 shows several example samples used to train disease object detection.

**TABLE 2:** Number of bounding boxes for the training and testing of disease objects.

Name	First stage		Second stage	
	Bounding boxes		Extended bounding boxes	
	Training	Test	Training (Aug)	Test
Angular leafspot	818	265	6,162	265
Anthracnose (fruit rot)	188	57	1,424	57
Anthracnose (runner)	237	166	30,897	166
Blossom blight	1,906	265	18,182	265
Gray mold (fruit)	1,468	224	13,069	224
Leaf spot	2,353	497	14,627	497
Powdery mildew (fruit)	405	161	2,626	161
Powdery mildew (leaf)	1,764	371	14,313	371
Normal (flower)	–		967	92
Normal (fruit)	–		1,842	104
Normal (leaf)	–		10,984	1,066
Normal (runner)	–		31,191	452
Unknowns	–		3,830*	862
Total	9,139	2,006	150,114	4,582

*Second stage unknown training data prepared for lazy classifier K-NN to find the unknown, which is unseen while training the feature extractor (ResNet).

### Training Deep Metric Learning (DML) With Softmax and K-Nearest Neighbor (*K*-NN) Classifier

For the DML with the softmax classifier, we used the same training/test dataset that we used for the first object detection stage. To increase the training data, the same augmentation techniques were taken as in the first stage. The increased number of images of the extended bounding box can be seen in column 3 of Table 2, which include additional normal (flower, fruit, leaf, and runner) objects so that the embedded features can be learned differently from disease symptoms. In addition, the training of the CNN backbone started from the weight pretrained by the ImageNet dataset. We trained the network in 300 epochs with a batch size of 128. The learning rate was set to 1e^–5^ and 1e^–4^ for the backbone network and the classifier head, respectively. We used the Adam optimizer and the semi-hard margin sampling threshold set to 0.01.

**FIGURE 6:**
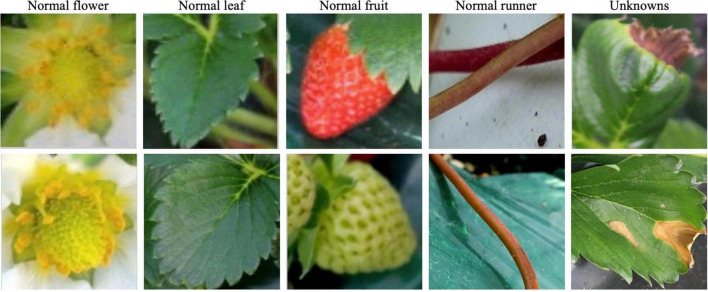
Sample images of normal leaf, fruit, flower, and runner, with unknowns.

After training the DML, we took the 256-dimensional features for reference images, which include eight known strawberry diseases with normal leaf, fruit, runner, and flower, and unknown diseases, and selected samples are shown in Figure 6. The unknowns are not included in the training by the DML with the softmax classifier for the second stage, but the embedded features for unknowns are taken to build the *K*-NN classifier after training.

### Results of Disease Detection

Table 3 presents the final results that explain the effect of post-filter. The results of the first stage of FPN-based Faster R-CNN and the second stage of classifiers are measured by average precision (AP) for each disease, and overall performance is obtained in mAP. The detection performance is found to be better for anthracnose (fruit rot) and blossom blight but comparatively worse for angular leafspot, anthracnose (runner), and powdery mildew (leaf). This is why the appearance of symptoms can be confused with other diseases (e.g., leafspot) or illumination reflecting on the leaves. In addition, the disease on the thin and long runner does not have sufficient resolution for it to be discriminated well, as is the case in the example of the anthracnose (runner).

**TABLE 3:** Final results of known disease detection for the test data.

Name	AP		
	Faster R-CNN	+ Softmax classifier	+ Comb. w. *K*-NN classifier
Angular leafspot	0.853	0.923	0.922
Anthracnose (fruit rot)	0.977	0.992	0.991
Anthracnose (runner)	0.865	0.885	0.883
Blossom blight	0.985	0.983	0.986
Gray mold (fruit)	0.881	0.905	0.904
Leaf spot	0.932	0.940	0.944
Powdery mildew (fruit)	0.924	0.958	0.956
Powdery mildew (leaf)	0.830	0.822	0.844
mAP	0.906	0.926	0.928

When the DML with the softmax classifier was added to the object detection stage, the mAP increased approximately 2%, as can be seen in the third column of Table 3, but two diseases showed a slight degree of performance decrease: blossom blight and powdery mildew (leaf). In our conjecture, this is caused by the dislocation of bounding boxes enclosing the disease symptom in the first object detection stage, even though the enlarged bounding box is fed into the post-filter. In this case, there could be an erroneous decision in the second stage because the input image has never been experienced in the training phase.

**FIGURE 7:**
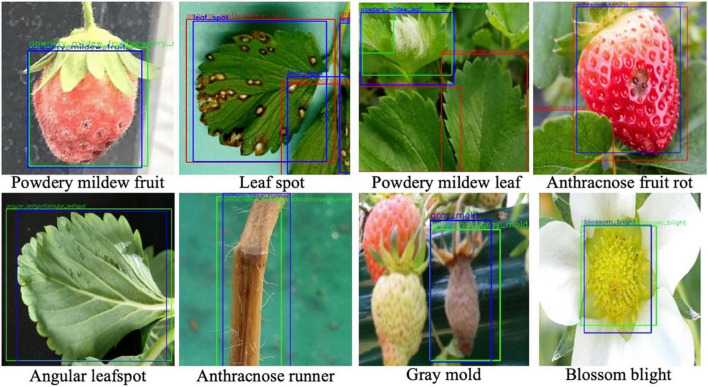
Disease detection results from object detection and post-filter. Objects are annotated by different box colors and prediction labels. Blue bounding boxes are the ground truth annotation. Detected bounding boxes are labeled by “A| B” with two categories; “A” is the prediction result in the first stage, after which the detected area is cropped into patches and sent to the DML and given prediction label B. Green boxes mean prediction labels A and B are the same, otherwise they are red.

However, when the two decisions from the softmax and *K*-NN classifiers are combined by Eq. (5), the AP performance for each disease was increased. As listed in the last column of Table 3, the effect of the combined decision was not significant, but there was a consistent performance increase for all diseases. Figure 7 shows the disease detection results from the Fast R-CNN object detection followed by post-filter. A red box means a different prediction result in object detection and DML post-filter, and a green box means the two decisions are the same. The object detector finds potential objects well if the detected object is distinct from the background. However, the detector may give a false prediction label if the background is complex. For example, for the “powdery mildew leaf” in Figure 7, the network misdetected a normal leaf as a powdery mildew leaf, and the difference between these two categories is that the disease-infected leaves are covered in snow-white fungus, but the reflection of light on leaves shares similar features. The DML post-filter focused on the local context and successfully corrected the false detected object.

**FIGURE 8:**
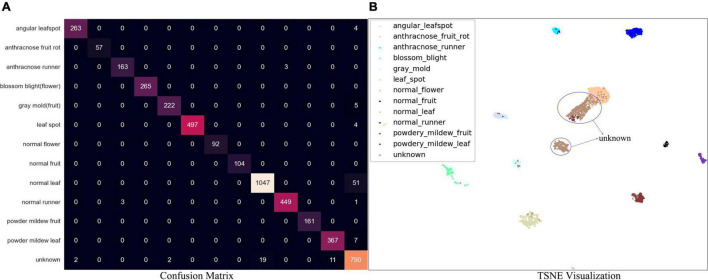
**(A)** Confusion matrix of DML-based *K*-NN classifier. **(B)** TSNE visualization result for test data.

For separated DML followed by the *K*-NN classifier, the performance has been visualized by a confusion matrix, which is shown in Figure 8A. Note that the separate stage can be used for the classifier of ROI of the symptoms. For example, a picture of disease-like symptoms can be taken and a manual ROI can be denoted without using an automatic disease detection model such as Faster R-CNN, after which its class can be obtained from this separate *K*-NN-based classifier. The overall accuracy of the separate *K*-NN classifier was 97.7% for the test data in the last column of Table 4, the summarized confusion matrix. In Table 4, the average recall and average precision were 96.7 and 97.7%, respectively. Again, a few instances of angular leafspot, gray mold (flower), and powdery mildew (leaf) were misclassified as unknowns. In addition, several normal (runners) were misclassified as anthracnose disease. Some unknown symptoms were confused with disease classes including angular leafspot, leafspot, gray mold (fruit), powdery mildew (leaf), and normal parts. Note that it is difficult to discern leafspot and angular leafspot from disorders on a leaf. For the same reason as in the object detection, there were several instances of confusion of disease classes of gray mold (flower), powdery mildew (leaf), and anthracnose (runner).

**TABLE 4:** Reduced confusion matrix.

Category	Diseases	Normal	Unknowns	Recall (%)
Diseases	1,999	3	4	99.7
Normal	3	1,692	19	98.7
Unknowns	20	52	790	91.7
Precision (%)	98.9	96.9	97.2	97.8(Accuracy)

Figure 8B shows the t-SNE of the embedded features after DML. It is evident that almost all the classes of known diseases and normal parts are well separated, but the classes that confuse (Figure 8B and Table 4) are slightly overlapping, as shown in Figure 8B.

### Final Field Test With Unseen Data

**FIGURE 9:**
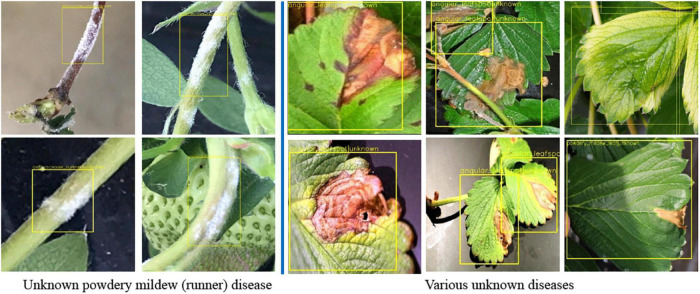
Detected unknown diseases.

To validate the proposed scheme, strawberry images were captured from three greenhouses at different locations, and we used these images to construct the dataset as in Table 5. Note that only six known diseases are included, because at that time, leafspot and anthracnose (fruit rot) were hard to find. In the table, powdery mildew (runner) can be treated as unknown, because it was not considered in the training of any building block of our scheme. Table 6 presents the mAP results of known diseases. It can be seen that the overall performances are increasing from the first object detection to the final combined decision of the softmax and *K*-NN classifiers. For unknown powdery mildew (runner), 19 images were detected with the proper bounding box out of 24 images. As shown in the left part of Figure 9 (left), all the diseases were detected as anthracnose (runner) in the first object detection stage but corrected to unknowns in the *K*-NN classifier. Moreover, as shown in the right part of Figure 9, the disorders on the leaf are corrected to unknowns in the *K*-NN classifier after having been wrongly detected in the first stage as one of the leaf diseases.

**TABLE 5:** Strawberry images for field testing.

Location	Disease	# of images
Chugbuk chongju	Blossom blight	24
Chungnam non-san	Angular leafspot	36
Jeonbuk wanju	Blossom blight	167
	Gray mold (flower)	54
	Anthracnose (runner)	47
	Powdery mildew (fruit)	63
	Powdery mildew (leaf)	42
	Powdery mildew (runner)	24*
Total		457

*Trained system has never experienced disease.

**TABLE 6:** Field test results of known disease detection.

Disease	BBox	Performance (AP)		
		Faster R-CNN	Faster R-CNN + softmax classifier	Faster R-CNN + *K*-NN combined decision
Angular leafspot	75	0.934	0.943	0.939
Blossom blight (f)(flower)	195	0.994	0.993	0.996
Anthracnose runner	161	0.853	0.866	0.913
Gray mold (fruit)	63	0.949	0.958	0.951
Powdery mildew fruit	48	0.881	0.915	0.931
Powdery mildew leaf	78	0.848	0.902	0.893
Total	620	0.909	0.930	0.937

## Conclusion

This study has proposed a simple but effective strawberry disease detection scheme with unknown diseases that can produce reasonable performance. In the proposed scheme, the known strawberry diseases are better detected with DML-based classifiers, as are the unknown diseases that have certain symptoms. We have assumed that, in the training process, the unknowns are partly known. The pipeline of our proposed scheme consists of two stages: the first is an object detection stage with known disease classes, while the second is the DML-based post-filtering stage. The second stage has two different types of classifiers: softmax classifiers for only known diseases and the *K*-NN classifier for known and unknown diseases. In training the first stage and DML-based softmax classifier, we have only used the known samples of strawberry diseases. Then, we included the known unknown training samples to construct the *K*-nearest neighbor classifier. The final decision for known diseases has been made based on the combined results of the two classifiers, while unknowns have been detected from the *K*-NN classifier.

The experimental results showed that the DML-based post-filter was effective at improving the performance of known disease detection in terms of mAP. Furthermore, the separate DML-based *K*-NN classifier provided high recall and precision with respective average values of 96.7 and 97.7%, showing it could be exploited as an ROI classifier. For the real field data, the proposed scheme achieved a high mAP of 93.7% to detect seven classes (six known diseases and one unknown) of strawberry disease, and it also achieved reasonable detection results for unknowns. These results imply that the proposed scheme can be applied to find disease-like symptoms due to real known and unknown diseases or disorders for any kind of plant, including strawberry.

## Data Availability Statement

The original contributions presented in the study are included in the article/supplementary material, further inquiries can be directed to the corresponding author.

## Author Contributions

JL supervised the whole project and wrote the original draft of the manuscript. KJ responded to collect the data resource and organized the database. JY performed the experiment and statistical analysis. All authors contributed to manuscript revision, read, and approved the submitted version.

## Conflict of Interest

The authors declare that the research was conducted in the absence of any commercial or financial relationships that could be construed as a potential conflict of interest.

## Publisher’s Note

All claims expressed in this article are solely those of the authors and do not necessarily represent those of their affiliated organizations, or those of the publisher, the editors and the reviewers. Any product that may be evaluated in this article, or claim that may be made by its manufacturer, is not guaranteed or endorsed by the publisher.
